# Large-scale binding affinity calculations on commodity compute clouds

**DOI:** 10.1098/rsfs.2019.0133

**Published:** 2020-10-16

**Authors:** S. J. Zasada, D. W. Wright, P. V. Coveney

**Affiliations:** Centre for Computational Science, University College London, 20 Gordon Street, London WC1H 0AJ, UK

**Keywords:** molecular dynamics, free energy, cloud, workflow, drug binding

## Abstract

In recent years, it has become possible to calculate binding affinities of compounds bound to proteins via rapid, accurate, precise and reproducible free energy calculations. This is imperative in drug discovery as well as personalized medicine. This approach is based on molecular dynamics (MD) simulations and draws on sequence and structural information of the protein and compound concerned. Free energies are determined by ensemble averages of many MD replicas, each of which requires hundreds of cores and/or GPU accelerators, which are now available on commodity cloud computing platforms; there are also requirements for initial model building and subsequent data analysis stages. To automate the process, we have developed a workflow known as the binding affinity calculator. In this paper, we focus on the software infrastructure and interfaces that we have developed to automate the overall workflow and execute it on commodity cloud platforms, in order to reliably predict their binding affinities on time scales relevant to the domains of application, and illustrate its application to two free energy methods.

## 1. Introduction

The accurate prediction of the binding affinities of ligands to proteins is a major goal in drug discovery and personalized medicine. A wide range of techniques are available to compute binding affinities (also known as binding free energies) from atomistic simulation. One of the most popular and accurate approaches is alchemical methods. They employ molecular simulations—molecular dynamics (MD) or Monte Carlo (MC)—of unphysical, alchemical intermediate states that attenuate the interactions of the small molecule with its environment [1].

The large changes which need to be accounted for in calculating absolute binding free energies (the ‘appearance’ or ‘disappearance’ of a ligand) are often slow to converge and consequently extremely computationally expensive. In order for simulations to have an impact in industrial or clinical decision making processes calculations must not only be accurate, precise and reliable but also complete in a short period of time. An attractive alternative to absolute calculations is thus the calculation of the difference in binding affinity between two systems (known as the relative binding free energy). In this paper, we employ ensemble MD-based thermodynamic integration with enhanced sampling (TIES) protocols [2] to produce rapid and reliable calculations of relative binding free energies, and present the binding affinity calculator (BAC),^1^ a system to manage such simulations.

Large-scale computing resources, both clouds and supercomputers, are revolutionizing the scientific investigations that can be performed *in silico*. The sheer computing power available to researchers makes new scientific investigations possible. Efforts to simply scale a single monolithic code linearly to the full production partiton of a supercomputer unnecessarily limits the range of problems that can be tackled to a very small set of algorithms with appropriate scalability characteristics, and effectively excludes cloud resources made up of losely coupled compute node. However, both clouds and supercomputers can be fully exploited by hybrid applications, for instance composed of ensembles of tightly coupled simulations running at smaller core counts.

The purpose of the present paper is to describe the software and associated computing environments necessary to perform rapid, accurate, precise and reproducible ligand–protein binding free energy calculations in an easy to use manner. In the past few years, we have shown how this may be done based on ensemble averaging of a sufficient number of independent classical MD simulations. Our approach uses two protocols known as ‘TIES’ and ‘enhanced sampling of MD with approximation of continuum solvent’ (ESMACS) [3]. ESMACS [4] is centred on the molecular mechanics Poisson–Boltzmann surface area (MMPBSA) method, while TIES is built around thermodynamic integration.

The protocols are complementary in that ESMACS is an absolute free energy method able to estimate binding affinities for highly diverse ligands of varying charges, whereas TIES is particularly suitable for estimating relative free energies of pairs of similar (congeneric) compounds and/or mutated protein sequences.

Knowledge of the free energy of binding of a molecule (a ligand) with a target protein, the binding affinity, is of central importance in drug discovery and drug design, as well as personalized medicine. Within the pharmaceutical industry, the binding affinity is the single most important quantity in the early stages of drug discovery, and is repeatedly required in later stages of drug design and optimization. In the more forward looking field of personalized medicine, drug treatment will increasingly be based on selecting the appropriate drug for a patient based on his or her genotypic and phenotypic profiles. In these situations, it is necessary to be able to discriminate the binding of ligands with sequence-specific variants of the same protein. Not only must such binding affinity predictions be made accurately, the protocols used must be reproducible if they are to confer the level of reliability required to be adopted within industry and by regulatory authorities concerned with public healthcare. The speed at which such calculations are made is also of the essence to ensure that the predictions are actionable in the former case to direct experimental drug discovery programmes, and in the latter to ensure treatments are timely.

Computational biomedicine [5] is one field successfully exploiting the potential offered by hybrid applications running on large-scale computational resources. Computational biomedicine uses computer-based simulation of biological systems to support, for example, the drug discovery and selection process. The apotheosis of such approaches is personalized medicine, which seeks to develop a new medical approach, in which data obtained from a patient, such as genomic information, are used to customize health management.

The natural variations in human genes are known to influence the risks of developing many diseases, and the response to a particular treatment. At clinical level, a disease usually appears to be of a single type, whereas at a molecular level, it could be classified as one of several sub-types, based on distinctive signatures of gene sequences, expressions and pathways. The treatment of a disease can then be tailored for the individual patient, based on the molecular classification made. The individual treatment of many cancers, for example, usually uses targeted radiotherapy to kill malignant cells, and/or tumour-growth inhibitors in an attempt to selectively target and kill tumour cells. This often involves a scheme called ‘targeted therapy’, where anti-cancer drugs are directed against cancer-specific molecules and signalling pathways. These are designed to interfere with a specific molecular target, usually a protein that plays a crucial role in tumour cell growth and proliferation.

Such approaches are also applicable to the drug discovery process where, given sufficient computational resources, potentially thousands of compounds can be screened against a target pathogen. Simulation offers the possibility to assess the effectiveness of certain courses of treatment before they are administered using a patient-specific model, in order to choose the best. This can be done only at a fully molecular level, based on the sequence and structure of the protein, drug(s) and the molecular inhibitors.

## 2. Existing approaches

There are several software tools which attempt to automate the free energy calculation method(s) based on atomistic MD simulations. Among them FESetup [6] and free energy workflow (FEW) [7] automate the set-up of alchemical relative free energy method thermodynamic integration (TI) [8] and endpoint methods MMPBSA [9] and linear interaction energy (LIE) [10]. On the other hand, free energy perturbation/replica exchange with solute temporing (FEP/REST) [11] attempts to enhance the free energy perturbation (FEP) method, another alchemical relative free energy method [12]. Forcefields used include AMBER [13] and OPLS [14]. A common feature of all these approaches is the use of the traditional one-off simulation technique to sample the phase space irrespective of the method used for the calculation of free energy. This limits them all to unreliable free energy predictions unlike BAC, since each individual simulation in fact behaves as a random process, and is not reproducible [15]. In particular, FESetup does not completely support alchemical codes with the dual topology scheme, while the time frame for performing TI calculations using FEW is of the order of several days.

## 3. Binding affinity calculator workflows

The BAC workflow comprises a number of distinct components which offer a great deal of flexibility in how they are deployed on a high-performance or cloud computing infrastructure. For a full discussion of the steps required to prepare a simulation using BAC, the reader is directed to [16], but briefly they are described below.

For a particular ligand–protein combination, BAC initially runs a set of job set-up scripts to create the necessary input files for a set of simulations, by taking a model manually prepared by a researcher and customizing it.

Next, these model data are staged to an appropriate computational resource and an MD code is used to run a number of equilibration steps to prepare the model, followed by a number of nanoseconds of simulation and free energy calculation, which in reality take several hours of wall-clock time per simulation. In order to be able to bound errors effectively, often hundreds of such simulations are run in parallel. Once the simulation and free energy calculation chain is complete, the output data (measured in giga- to terabytes) are post-processed to extract the parameters of interest. Typically, this workflow needs to be run in its entirety several times (once for each of the compounds and/or sequence-specific proteins involved) in order to generate a ranking of the binding affinities obtained.

To run this workflow in an automated fashion, a comprehensive software and hardware infrastructure is required, which takes care not only of executing the necessary computations but also managing and staging the data pertaining to the ligand–protein system and simulation output data. Infrastructure components required include systems to store and manage the data, and computational platforms to execute the calculations and analyse the data arising.

Our ESMACS and TIES approaches decompose the workflow of a complete calculation into three main components: (a) preparation of a bimolecular model, which includes generation of force field parameters if needed, and a simulation-ready molecular model, (b) the MD calculation, which consists of ensemble MD runs, and (c) the post-processing analysis of the model through which the binding affinity predictions are calculated. The workflow consists of a sequence of connected steps, including performing the three individual components and data transferring between various computational resources. The whole process can be performed automatically by a single command, or the discrete tasks can be executed separately, on various computational resources. A series of scripting commands, which together constitute the BAC builder, are used to set up an MD simulation and create the necessary input datasets to run MD simulations on a supercomputer, to post-process the simulation trajectories and produce results and to transfer the data between various resources.

To have rapid, accurate, precise and reproducible prediction of ligand–protein binding free energy, a protocol has been developed in which an ensemble of MD simulations are performed for each protein inhibitor (also known as a complex). Our previous studies have shown that an ensemble of 25 replica simulations of 4 ns length each are required for each ligand–protein complex, and a free energy calculation is performed at uniformly chosen snapshots from each simulation trajectory. This leads on to the order of a thousand snapshots generated and analysed for each system. The BAC workflow automates the complexity of running and marshalling these simulations, and collecting and analysing data. BAC depends on the ability to perform hundreds of separate parallel simulations on a high-performance computing (HPC) platform, each of which might require 32–500 cores depending on the system.

## 4. Requirements

On a present-day high-performance or cloud computing platform with many thousands of cores, in the time it takes to run one calculation, we can do as many ensembles as are required, meaning there is no need for extended wall-clock time; hence the ability to predict on a clinical time scale is now entirely possible, given sufficient resources. The problem is the availability of such resources. Typically, access to the largest scales of HPC is available primarily to academic researchers via a competitive grant proposal process, or to government-funded institutions. Furthermore, in normal settings of this kind jobs are scheduled through a batch queuing process, meaning that no guarantee can be given as to when any individual job will be run.

The nature of BAC workflows means that the computations can be decomposed into ensembles of smaller MD simulations. While each simulation requires typically 32–64 tightly interconnected compute cores, the overall ensemble of simulations behaves like an ‘embarrassingly parallel’ job, with no requirement for inter-process communication. As such, these types of ensembles map very closely to the architectures of commercially available compute clouds, which make available single compute nodes of up to 128 CPU cores and nodes with multiple GPUs. The on-demand nature of commodity cloud means that such resources can be called up when required and only the resources used are paid for.

The BAC workflow automates much of the complexity of running and marshalling these simulations, and collecting and analysing data. Even though we have taken steps to automate model building, execution and analysis of binding affinity calculations, the process of running a BAC simulation manually is necessarily complicated. For that reason, we have developed the ufBAC system, where ‘uf’ stands for ‘user friendly’ and takes a *cloud-first* approach.

ufBAC has been developed to satisfy two broad use cases:

(i) The end user from academia/industry who wants to use ufBAC as a black box, to generate results from a pre-built model, having those results presented in the form of an emailed/downloaded report. The user needs to be able to monitor their simulations and share results with others. The click of a button should be all that is required to build, execute and analyse the user’s chosen model. Once the simulation/study has completely executed the user is notified/sent a report summarizing the results.
(ii) The power user who has the same requirements as the regular user, but with finer-grained control over all aspects of the simulation building and execution process. Additionally, the power user requires access to individual simulation output files and statistical packages to process output results.

In turn, these use cases have driven the development of the ufBAC system as a web-based tool that groups of researchers can use to collaborate on the development and execution of binding affinity models, which exploit significant HPC power by spawning multiple simulation replicas to generate the statistics required to ensure the results are accurate and reliable.

ufBAC is a web portal-based interface to the BAC, which allows a user to build models of molecule–compound binding, and execute and analyse multi-replica MD simulations using the model. The binding affinity may be calculated by ESMACS and TIES methods.

Porting the workflow to commodity cloud platforms mandates a further requirement: that the application is portable. This means that the port of the BAC workflow from HPC to cloud systems must be done in such a way that it can be run on any commodity cloud with minimal changes. The implication of this is that the platform-specific offerings of a particular cloud cannot be heavily used, to avoid vendor lock-in.

## 5. Architecture

Selecting a cloud computing platform involves a trade-off between flexibility and vendor lock-in. While the basic commercial offering of the major cloud vendors is broadly similar, each vender offers its own value-added services on top of the basic platform. The more of these services that are used, the closer you are tied to a particular cloud vendor and the harder it is to switch platforms. We have taken an approach to limit the possibility of vendor lock-in by building on a common set of components that allow us to easily port our applications between clouds, namely Docker and Kubernetes.

In order to keep the BAC workflow components portable between cloud platforms, ufBAC wraps each component of the workflow inside a Docker container. Docker is a system widely supported on commodity compute clouds that creates a lightweight Linux virtual machine with an externalized kernel. This allows each component to be executed in a controlled environment that does not depend on installing the required packages on each target compute cloud. Different components of the BAC system are decomposed and wrapped in Docker containers, allowing them to be run independently. A full ESMACS workflow, for example, consists of running all of the ESMACS components in order. If parts of the workflow are performed manually (such as model building), then running the corresponding Docker container can be omitted.

Each Docker container wrapping an application contains all of the libraries and software tools required to run the application. The Docker containers are stored in a Docker registry, from which they can be downloaded and run.

**Figure 1.**
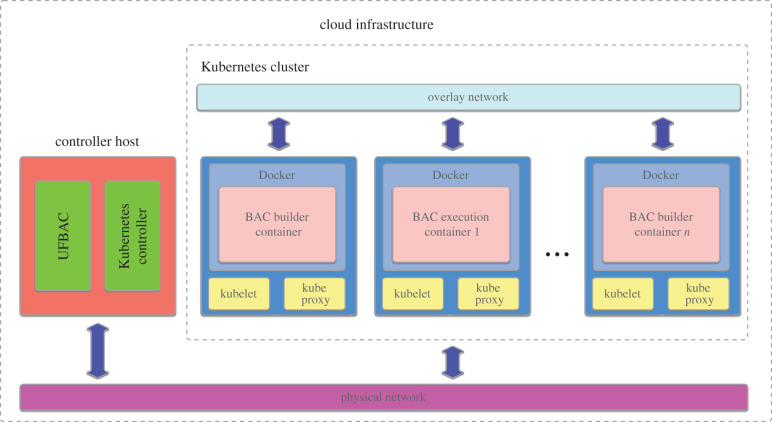
Kubernetes provisions a virtual cluster that allows independent application components wrapped in Docker containers to be executed within a secure virtual network.

In order to run Docker containers, a Kubernetes cluster must be provisioned on the target cloud platform. This cluster is somewhat like a loosely coupled HPC cluster, comprising nodes of a specific type. Kubernetes is able to download and run instances of a Docker container from a Docker repository, relieving the user of the need to manually provision cloud resources, as shown in figure 1. Kubernetes is available as a managed service on all the major cloud platforms, meaning a Kubernetes cluster can be deployed trivially and scaled as required on Azure, AWS and Google Cloud Platform. Doing so also takes care of configuring requisite network connections and access patterns. Costs are incurred as long as a Kubernetes cluster is provisioned, whether any applications are running or not. For this reason, ufBAC provisions Kubernetes clusters on demand, and when the compute is complete the associated cluster is deleted. This removes all components of the cluster that have been automatically created, so they are not billed for.

Each run on the BAC workflow requires a storage container to be created, to contain the input and output data generated by the different stages of the workflow. This is the only cloud-specific service used by a BAC deployment, using Azure Blob Storage when deployed on Azure for example.

The nature of Docker means that containers can be built upon other containers. This assists us in keeping the core application components generic, for deployment on any commodity cloud, then building cloud-specific containers for each target platform, based on the generic containers, which include code required to access the storage services of the specific cloud in question.

The process of manually running a BAC workflow on Azure cloud follows the pattern:

(i) Create a Blob Storage container,
(ii) Upload tarball containing a PDB and associated files,
(iii) Deploy a kubernetes cluster,
(iv) Run the builder,
(v) Run a sanity check to test no zero length files are created,
(vi) Run NAMD with or without MMPBSA,
(vii) Optionally run NMODE,
(viii) Run analysis to gather results.

ufBAC enables BAC to be run via a Software as a Service model, hiding from the user the complexities of the command line tools and API calls used to build models, executing them on HPC resources, and analysing the results. ufBAC is intended to plug in to a range of computational back ends, with a cloud-first approach preferred.

**Figure 2.**
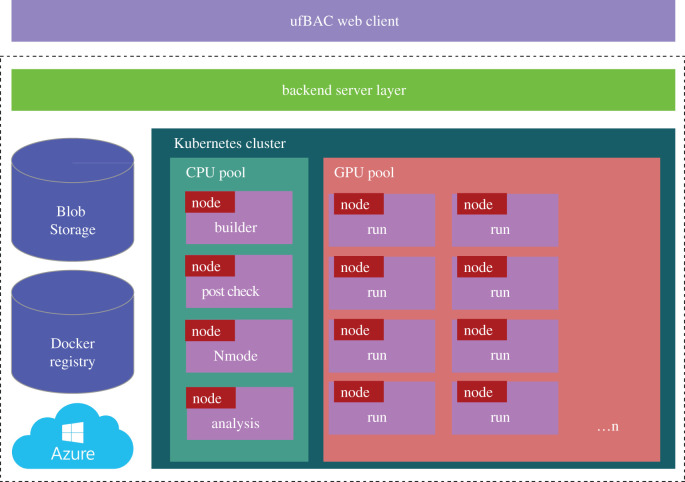
The multi-layered architecture of the ufBAC system. The system comprises a web interface, through which the user interacts with it, server components to manage the execution of simulations and data, and the BAC tool itself, which effectively constitute a *Software as a Service* application, built on publicly available high-performance computer systems.

ufBAC seeks to unify the different components of the BAC workflow required to build models, execute them and analyse the results, into a single unified system with a single interface. In order to do this, it implements a multi-layered architecture, shown in figure 2. The first is the client layer, a user portal developed using Google Web Toolkit (GWT) [17]. The user interacts with the BAC system via their web browser. The use of GWT provides a mechanism to develop high-performance, low-overhead web interfaces developed using Java which are compiled into separate JavaScript/HTML and Java byte code components, with the former running inside the user’s browser (reducing server overheads) and interacting with the latter running inside a web application container such as Tomcat. It also means that new interfaces (designed for mobile devices for example) can easily be constructed, which make use of the common functionality provided by the server side part of the client.

The purpose of the ufBAC system, and the portal in particular, is to make the process of running complicated simulation workflows that rely heavily on HPC as simple as possible, improving usability by moving the user away from the command line towards a user-friendly cloud-style application. The ufBAC web portal follows a conventional design. The left-hand side of the interface contains a menu bar that allows the user to access the various features of the application. The top bar of the website displays user notifications (for example that a set of simulations have finished running). The main content panel gives access to the features of the application and allows users to control running simulations, create and execute new models and analyse data.

The server layer comprises the server components of the user portal, which are responsible for user login, state management and presentation. This layer either uses a cloud platform provided REST API to set up and control the required Kubernetes infrastructure or, where BAC is run on local, non-cloud resources, builds on an application hosting environment (AHE) [18]. AHE is designed to hide much of the complexity of dealing with HPC resources from users of such systems, allowing them to interact with applications rather than machines. The nature of AHE means that it can be used as a single interface to a wide variety of resources, ranging from those provided at a departmental or institutional level, through regional and national, to international federated cyberinfrastructures of supercomputers. AHE is used to manage the execution of BAC layer components.

The third layer of the architecture, the BAC layer, wraps around existing BAC workflow services. These comprise the BAC Builder, a tool to construct a simulation model, BAC Execute, a set of scripts used to generate simulation replicas, stage data and execute models and free energy calculations, and the statistical analysis component used to analyse simulation output data and automatically generate figures and results tables.

## 6. ufBAC in practice: ROS1

ROS1 is a transmembrane receptor protein tyrosine kinase which has been identified as playing an important role in a range of pathologies (including glioblastoma, colorectal cancer, ovarian cancer and non-small cell lung cancer). This has led to the kinase domain becoming a promising target for drug discovery. Janssen provided a dataset of 21 neutral congeneric ligands designed to target ROS1. In this study, we selected 17 pairs of ligands for use in evaluating the ability of TIES-based relative binding free energy calculations to reproduce experimental ranking between the compounds. All the work was executed on the Microsoft Azure cloud, using the cloud deployed BAC described in the previous sections.

**Figure 3.**
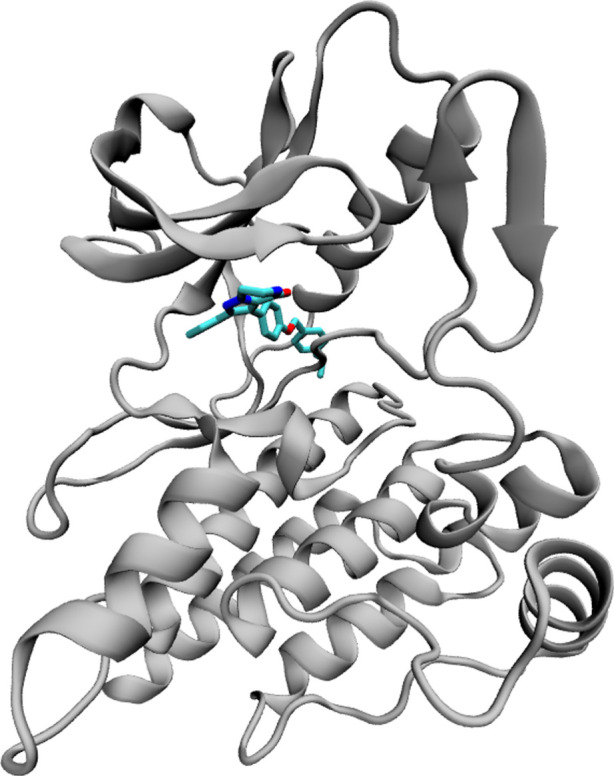
Crystal structure of the ROS1 tyrosine kinase (shown in cartoon representation) bound to JNJ-54192398 (depicted in chemical representation).

A structural model of the ROS1 kinase domain bound to compound JNJ-54192398 was provided by Janssen for the project based on an X-ray structure determined in-house at 2.64 Å resolution (figure 3). A loop region in the sequence, Lys2117–Gly2121, was not resolved in this crystal and was extracted from a non-disclosed in-house ROS X-ray structure and merged with the main structure.

## 7. Simulation methodology

TI is well known in the literature [19]. The relative binding affinity of two ligands L1 and L2 is calculated by considering an alchemical transformation between them connected through intermediate states defined by introducing a coupling parameter *λ*, such that at *λ* = 0 the system corresponds to ligand L1 (initial state) and at *λ* = 1 the system corresponds to ligand L2 (final state). The total energy of the system is taken to be its potential energy (*V*). The energy of the system can be defined as

7.1 $$V(\lambda ,x)=(1-\lambda )V_{1}(\lambda ,x)+\lambda V_{2}(\lambda ,x),$$

where *V*_1_ and *V*_2_ are the potential energies of ligands L1 and L2 calculated using a chosen molecular mechanics force field.

In this paper, we use the TIES method [2] to calculate the absolute or relative free energy corresponding to an alchemical transformation (Δ*G*_alch_). We denote the alchemical coupling parameter as *λ*. Δ*G*_alch_ is given by the equation

7.2 $$\Delta G_{\mathrm{alch}}=\int_{0}^{1}{\left\langle \frac{\partial V(\lambda ,x)}{\partial \lambda }\right\rangle }_{\lambda }\;\text{d}\lambda ,$$

where 〈…〉_*λ*_ denotes an ensemble average in state *λ* and 〈∂*V*(*λ*, **x**)/∂*λ*〉 is the derivative of the hybrid potential function. For 〈∂*V*/∂*λ*〉 to be calculated, an ensemble of MD simulations is run at each window corresponding to an intermediate value of *λ*. We evaluate equation (7.2) using a stochastic integration method because the integrand comprises points that are Gaussian random processes. In TIES analysis, the integral in equation (7.2) is treated as a stochastic integral, and the associated uncertainty is calculated accordingly, as described by Bhati *et al.* [2].

The thermodynamic cycle approach is employed to calculate the relative binding affinities ΔΔ*G* between these two ligands associating with a protein using the following equation:

7.3 $$\Delta \Delta G=\Delta G_{1}-\Delta G_{2}=\Delta G_{\mathrm{alch}}^{\mathrm{aq}}-\Delta G_{\mathrm{alch}}^{\mathrm{bound}},$$

where Δ*G*_1_ and Δ*G*_1_ are the binding free energies of ligands L1 and L2, respectively. $\Delta G_{\mathrm{alch}}^{\mathrm{aq}}$ and $\Delta G_{\mathrm{alch}}^{\mathrm{bound}}$ are the free energy differences associated with the alchemical transformation of ligand L1 into L2 in free and bound states, respectively.

### 7.1. Model building

The simulations for each protein–ligand pair are initiated from the provided ROS1 crystal structure with the co-crystallized compound replaced with the appropriate hybrid ligand description. For each ligand pair, a hybrid topology must be created from independent topologies created for each ligand. Starting coordinates for each ligand are generated through the use of the Template CoMFA flexible alignment tool with the co-crystallized compound as a reference. This tool assigns ligand torsion angles with the value found for the matching torsion angles in the reference ligand. If no matching torsion is found, topomeric folding rules are applied. The single ligand parameterizations were created using the standard BAC protocol [16]. This procedure involves the optimization of the provided structure via Gaussian 98 using the Hartree–Fock method and 6-31G** basis functions. The partial atomic charges were then assigned using the restrained electrostatic potential (RESP) procedure, which is part of the AMBERTools package. The remaining force field parameters were described using the general AMBER force field (GAFF) [20]. The TIES approach relies upon the creation of hybrid ligand topologies that combine a common core with ‘disappearing’ and ‘appearing’ regions which contain the unique elements of the initial and final ligands in the alchemical transformation. The creation of the hybrid ligands in this study was performed using a recently developed extension to the BAC based on the maximum common substructure (MCS) functionality of the RDKit library (www.rdkit.org). Initial and final ligand geometries, connectivity and charges are taken from the AMBER parameterizations as used in ESMACS. The hybrid topology generator (github.com/dww100/ties_hybrid_topology_creator) first identifies the common chemical elements of the ligands excluding any incomplete rings, then further atoms (and rings of which they are part) are removed if the partial charge differs by more than a set tolerance (0.1 in the present study) between initial and final topologies. For atoms in which the charge difference is below this tolerance an average of the values for the two original ligands is used.

### 7.2. Thermodynamic integration with enhanced sampling protocol

We apply the TIES protocol as described in detail in Bhati *et al.* [2] The Amber package [21] was used for the set-up of the systems and analyses of the results, and the MD package NAMD2 [22] was used throughout the equilibration and production runs of all simulations, including the metadynamics.

In the current TIES study, the alchemical coupling parameter, *λ*, assumes the values 0.0, 0.05, 0.1, 0.2, 0.3, 0.4, 0.5, 0.6, 0.7, 0.8, 0.9, 0.95, 1.0. Van der Waals contributions were scaled linearly varying with *λ* across the full range (0 to 1). A soft core potential was used for the van der Waals interactions of all atoms in the alchemical space to avoid divergent potential energy due to the sudden appearance of atoms close to the end-points of the alchemical transformation, often called ‘endpoint catastrophes’ [23]. Moreover, the electrostatic interactions of the disappearing atoms were linearly decoupled from the simulations between *λ* values of 0 and 0.55 and completely turned off beyond that, whereas those of the appearing atoms were linearly coupled to the simulations from *λ* values of 0.45 to 1 and completely extinguished otherwise. For each *λ* value, five replica simulations are executed, resulting in 65 simulations being run for each ligand pair (the same protocol was employed for the ligand in both protein-bound and aqueous environments).

For each ligand pair, at each *λ* value, an ensemble simulation was performed with identical atomic coordinates for all replicas (for both the protein-bound and aqueous environments). Energy minimizations were first performed with heavy protein atoms restrained at their initial positions. The initial velocities were then generated independently from a Maxwell–Boltzmann distribution at 50 K, and the systems were heated up to and kept at 300 K within 60 ps. From this point, all systems were maintained at a temperature of 300 K and a pressure of 1 bar in an NPT (isobaric–isothermic) ensemble using the standard NAMD protocol of Langevin dynamics (with a damping coefficient of 5 ps^−1^) and a Berendsen barostat (compressibility of 4.57 × 10^−5^ bar^−1^ and relaxation time of 100 fs). A series of equilibration runs, totalling 2 ns, were conducted, while the restraints on heavy atoms were gradually reduced. Finally, 4 ns production simulations were run for each replica with five replicas used for each pair of ligands. Previous studies [24–26] have shown that the combination of the simulation length and the size of the ensemble provides a trade-off between computational cost and precision.

## 8. Results

**Figure 4.**
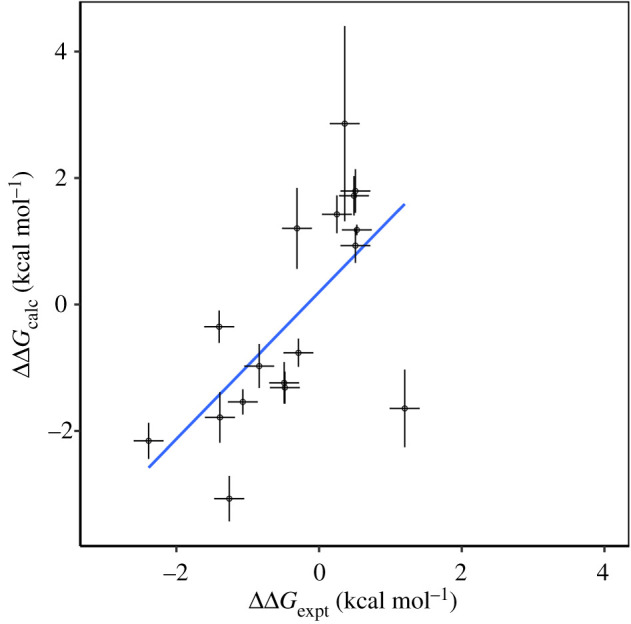
Comparison of the relative binding free energies of the 17 ligand pairs binding with ROS1 from experiment, ΔΔ*G*_expt_, and TIES calculations, ΔΔ*G*_calc_. The blue line shows a line of best fit.

We obtained reasonable agreement between the TIES calculated values (ΔΔ*G*_calc_) and experimental values (ΔΔ*G*_expt_) with Pearson correlation, *r*, of 0.64 (Spearman, *r*_*s*_ of 0.59) (figure 4).

This level of performance is lower than previous studies using TIES (which typically achieve *r* > 0.7) and is reflected in MSE of 1.65 kcal mol^−1^ (and a RMSE of 1.28 kcal mol^−1^). While this could be a result of the high plasticity of kinase active sites [27], ligand-specific reasons can be found for some of the results obtained. The worse performing pair is 5425 0638 to 5419 0110, in which an azetidine is replaced with an azinidine.

Small ‘ring’ moieties such as these can be inaccurately parameterized and our hybrid ligand creator does not have specific rules to handle this transform. The two transformations with errors of greater than 0.5 kcal mol^−1^ both involve the compound 54410707, suggesting that system-specific details play a significant role. Further investigation is required to identify the specific algorithmic and parameterization changes which may improve results in a way translatable to other systems. Our recent work in this area suggests that simple extensions of sampling (including those using enhanced sampling techniques employed in commercial solutions such as FEP+) will be insufficient [12]; see also [28].

## 9. Conclusion

Our system has been widely deployed and used to perform accurate, reproducible ligand–protein binding calculations. In the research setting, this has only been possible because of easy access to the large-scale computational resources required to perform the volume of calculations needed to generate accurate results. It has also been made possible because we have developed a software infrastructure to allow scientists to easily manage and access the simulation workflows, so that they can focus on the generation of results.

Our initial motivation for this work was to support patient-specific decision making, ensuring the approach is rapid, accurate and reliable. More recently, it has become clear that the methods can play a role in drug discovery too. However, the large-scale ‘community’ resources widely used in academia are not commonly available to commercial organizations, which has motivated the porting of the BAC to commodity cloud platforms, adopting a flexible architecture that avoids, as far as possible, lock-in to a particular vendor. The availability of our ufBAC platform, backed by elastic cloud computing infrastructure, has resulted in interest in the TIES and ESMACS approaches by several pharmaceutical companies, and a series of pilot projects with companies interested in using the techniques to speed up different aspects of their drug discovery process, one of which we have reported in this paper.

The flexibility of our approach, being able to deploy on a cloud or HPC infrastructure depending on the resources available while providing a consistent view of both types of infrastructure via our ufBAC interface, makes the execution of such studies very straightforward. The implementation described herein uses the data security and networking features provided out of the box by cloud providers such as Azure. Further work will be required to assess the security requirements of prospective users and ensure that sufficient security controls are put in place.

While the workflow system we have presented has necessarily been tailored to the free energy methodologies and related tools that are of interest to our research, the modular approach can be applied to other methods and tools with some effort. In order to do so, engineering effort would need to be invested in the development of BAC scripts to set up the necessary input filesets and Dockerfiles to containerize the required applications, as well as modifications to the interface to present the results.

## Endnote

1 The BAC workflow code is available at: https://github.com/UCL-CCS/BAC2; the BAC container code is available at https://github.com/UCL-CCS/bac-docker

## Data accessibility

This article has no additional data.
